# Expression level of neutrophil extracellular traps in peripheral blood of patients with chronic heart failure complicated with venous thrombosis and its clinical significance

**DOI:** 10.1186/s13019-024-02506-3

**Published:** 2024-03-15

**Authors:** Fang Liu, Qian Zhai

**Affiliations:** 1 Medical Lab, The Second Affiliated Hospital of Xi’an Medical University, Xi’an, 710038 Shaanxi China; 2 Department of Blood test, Xi’an Blood Center, Shaanxi Blood Center, No.407 Zhuque Street, Yanta District, Xi’an, 710061 Shaanxi Province China

**Keywords:** Neutrophil extracellular traps, Chronic heart failure, Venous thrombosis, Predictive value

## Abstract

**Objective:**

Previous studies have reported that neutrophil extracellular traps (NETs) have been identified to be involved in thrombosis, but the clinical value in chronic heart failure (CHF) patients with venous thrombosis is unclear. This study focused on the expression level of NETs in the peripheral blood of patients with CHF complicated with venous thrombosis and its clinical value.

**Methods:**

80 patients with CHF were included and divided into 2 groups according to the occurrence of venous thrombosis, and the expression levels of NETs in peripheral venous blood and lesion veins of the patients were detected through fluorescent staining. Myeloperoxidase-DNA (MPO-DNA) and citrullinated histone H3 (CitH3), markers of NETs, were detected by enzyme linked immunosorbent assay kit. The receiver operating characteristic (ROC) curve was used to analyze the value of peripheral venous blood NETs in the diagnosis of venous thrombosis in CHF patients, while the relationship between NETs in peripheral and lesion veins was analyzed by a unitary linear regression model.

**Results:**

The results showed that the concentration of NETs, MPO-DNA, and CitH3 in CHF patients combined with venous thrombosis was markedly higher than that in patients without venous thrombosis, and the concentration of NETs, MPO-DNA, and CitH3 in lesion venous blood was notably higher than that in peripheral venous blood. Binary logistics regression analysis showed that NETs in peripheral venous blood were an independent risk factor for venous thrombosis in patients with heart failure. The unitary linear regression model fitted well, indicating a notable positive correlation between NETs concentrations in peripheral and lesion veins. The area under the ROC curve for diagnosing venous thrombosis was 0.85, indicating that peripheral blood NETs concentration levels could effectively predict venous thrombosis in CHF patients.

**Conclusion:**

The expression level of NETs was high in the peripheral blood of CHF patients combined with venous thrombosis and was the highest in lesion venous blood. NETs levels in peripheral blood had the value of diagnosing venous thrombosis in CHF patients, and the concentrations of NETs in peripheral and lesion veins are markedly positively correlated.

## Introduction

As a complex syndrome, chronic heart failure (CHF) refers to a persistent state of heart failure, including stabilization, deterioration, and decompensation. CHF impacts the heart to supply oxygen to tissues, and is caused by structural and functional impairment of the heart [1]. A research study showed that there are about 13.7 million patients (1.3%) with heart failure among residents aged ≥ 35 years in China, and the prevalence of heart failure has increased by 44% over 15 years [2]. Alem reported that endothelial dysfunction is closely related to CHF, and endothelium is involved in regulating blood flow, coagulation, vascular permeability, and structure [1]. Endothelial dysfunction is an important factor causing venous thrombosis [3], and vascular endothelial dysfunction in CHF patients triggers a hypercoagulable state of the blood, making CHF patients a high-risk group for venous thrombosis. Venous thromboembolism (VTE) is a vascular disease with high morbidity and mortality, and its main clinical events are deep vein thrombosis (DVT) and pulmonary embolism (PE) [4]. Virchow described the pathogenesis of venous thrombosis as a triad, namely venous stasis, blood hypercoagulability and vascular wall damage [5]. Therefore, it is important to quickly identify whether CHF patients have venous thrombosis [6, 7].

Neutrophil extracellular traps (NETs) are meshwork structures composed of DNA histone complexes and proteins released by activated neutrophils that capture bacteria, fungi, protozoa, and viruses [8, 9]. NETs can be mixed with granules and cytoplasmic components such as neutrophil elastase (NE), citrullinated histone H3 (CitH3), and myeloperoxidase (MPO) to fight infections or cancer burden [10]. It has been shown that thrombosis is associated with NTEs and the activation of intrinsic and extrinsic coagulation pathways [11]. For one thing, the large amounts of circulating free DNA (cfDNA) in NETs impairs fibrinolysis, thus forming scaffolds that can bind platelets, red blood cells, fibrin, and coagulation factors to promote venous thrombosis [12]. In addition to cfDNA, other components of NETs can also exert procoagulant properties through extracellular histones, triggering platelet activation to aggregate and coagulate blood [13, 14]. For another, NETs promote both intrinsic and extrinsic coagulation pathways through the activity of neutrophil serine proteases. Neutrophil elastase and cathepsin G are also present on NETs and enhance tissue factor and factor XII-driven coagulation through proteolysis by tissue factor pathway inhibitors [15]. Excessive formation of NET may promote immune thrombosis and even lead to organ failure [16]. Several studies have provided a basis for NETs mediating the pathological process of thrombosis. Skendros et al. [17] confirmed elevated MPO-DNA complexes, tissue factor activity, and sC5b-9 levels in the blood of 25 COVID-19 patients. Tissue factor is highly expressed and NETs carrying active tissue factor are released in neutrophils, inducing thrombogenic activity in human aortic endothelial cells. In the investigation of the causes of immune thrombosis in acute respiratory distress syndrome (ARDS), Zhang et al. [18] found that the levels of tissue factor-rich NETs (MPO and CitH3) are notably elevated in the plasma of ARDS patients and mice, and that tissue factor-rich NETs accelerate the formation and progression of ARDS immune thrombosis. Li et al. [19] found the presence of NETs in colon tissue and high plasma NETs levels in patients with active inflammatory bowel disease (IBD) and demonstrated a tendency for NETs to drive thrombosis in patients with active IBD.

The above studies highlight the potential clinical significance of NETs in thrombosis, but the relationship between NETs and concurrent venous thrombosis in CHF patients is unclear. Therefore, this study investigated NETs, MPO-DNA, and CitH3 levels in the peripheral blood of CHF patients with or without venous thrombosis and the relationship between NETs, MPO-DNA, and CitH3 in peripheral and lesion venous blood of CHF patients with venous thrombosis, and elaborated on the clinical significance for detection of NETs.

## Methods

### Patient collection

80 patients with CHF and 40 healthy subjects with VTE were admitted to The Second Affiliated Hospital of Xi’an Medical University (hereinafter referred to as “our hospital”) from September 2021 to September 2022 were included. CHF patients’ inclusion criteria were as follows: Patients with complete clinical data; with a definite diagnosis of CHF according to the 2014 AHA/ESC/HRS guidelines; with venous thrombosis meeting the diagnostic criteria for VTE; aged over 18 years and had signed informed consent. Exclusion criteria included: Patients with genetics thrombosis-related diseases; with hepatic and renal dysfunction, severe infections; with systemic diseases such as rheumatic diseases, ulcerative bowel disease, and systemic lupus erythematosus; with poor physical condition or confusion, and unable to communicate properly. CHF patients were divided into a VTE group (*n* = 40) and a non-VTE group (*n* = 40) according to the occurrence of VTE. This study complied with medical ethics standards and was approved by the ethics committee of our hospital.

### Observation indexes

12 mL venous blood samples were collected from fasting peripheral venous blood of patients in the three groups and from thrombotic lesions of CHF patients in the VTE group respectively. The collected samples were centrifuged at 37 ℃, 3000 rpm for 15 min and the supernatant was stored in a -80 ℃ cryogenic refrigerator.

NETs levels in peripheral blood (VTE-peripheral vein) and lesion venous blood (VTE-disabled vein): Dilutions were prepared referring to the instructions of the calf thymus DNA kit (Sigma, Germany). 1.0 mL of ddH_2_O dilution was added into 1 mg of lyophilized calf thymus DNA standard. We mixed them evenly to prepare 1 mg/mL standard solutions, which were diluted to 200, 100, 50, 25, 12.5, 6.25, and 3.125 ng/mL. After rewarming, 100 µL of plasma was incubated with an equal volume of 1 µM Sytox Green fluorescent dye (Invitrogen, USA) in the dark for 5 min. The fluorescence values of the samples and standards were detected at 485 nm by a fluorescence plate reader (Biotek, USA). The standard curve was drawn with the standard concentration as the abscissa and the fluorescence value as the ordinate, and then the NETs concentration levels of the samples were calculated according to the standard curve.

Quantification of MPO-DNA complex and CitH3: MPO-DNA complex and CitH3 are markers of NET. Levels of MPO-DNA complex and CitH3 protein were quantitatively determined using MPO-DNA ELISA kit (Roche, Switzerland) and CitH3 ELISA kit (Cayman Chemical, USA) according to the manufacturer’s instructions and the methods in Zuo’s study [20].

### Statistical methods

SPSS 22.0 software was used for statistical analysis. Measurement data conforming to the normal distribution were expressed as mean ± standard deviation, and t-test was used for comparison between groups. Count data were presented as cases (%), and the comparison between groups was conducted by chi-square test. The independent risk factors of venous thrombosis in patients with heart failure were analyzed by binary logistic regression. A unitary linear regression model was used to verify the correlation between NETs concentrations in peripheral and lesion venous blood of CHF patients with venous thrombosis. The receiver operating characteristic (ROC) curve was for analyzing the predictive value of peripheral blood NETs levels in CHF patients with venous thrombosis. The area under the curve (AUC) and 95% confidence interval (95% CI), sensitivity, and specificity were calculated. Youden index was used to determine the diagnostic cut-off value. *P* < 0.05 was considered statistically significant.

## Results

### Baseline characteristics of patients

80 CHF patients were included in this study and divided into VTE and non-VTE groups, with 40 patients in each group, and the baseline characteristics of the 2 groups are shown in Table 1. The mean ages of the two groups in this study were 65.2 ± 10.6 and 64.2 ± 10.2 years. The mean heart rates were 72.2 and 70.1 beats/min, and the mean systolic blood pressures were 118.4 mm Hg and 118.5 mm Hg in the 2 groups. The mean left ventricular ejection fraction (LVEF) was 27.4% ± 4.8 in the VTE group, with 65.0% of patients having LVEF less than 30.0%. The mean LVEF was 27.3% ± 4.9 in the non-VTE group, with 67.5% of patients having LVEF less than 30.0%. The generally low LVEF in patients is due to CHF [21]. The median N-terminal prohormone of brain natriuretic peptide (NT-proBNP) values, a crucial indicator in the treatment of heart failure, were 1187 and 1183 pg/mL in the 2 groups, and the proportions greater than 1000 pg/mL were 75.0% and 77.5%. There were 21 (52.5%) patients with ischemic heart failure in the VTE group and 20 (50.0%) in the non-VTE group, and 19 (47.5%) and 20 (50.0%) patients with non-ischemic heart failure respectively. No significant differences were observed between the two groups for indicators including age, sex, weight, heart rate, systolic blood pressure, LVEF, and cause of heart failure (*P* > 0.05). The similar baseline characteristics made the 2 groups comparable.

**Table 1:** Baseline characteristics of chronic cardiac failure patients VTE and non-VTE at baseline

Characteristic	VTE (*n* = 40)	non-VTE (*n* = 40)	*P*
Age, years	65.2 ± 10.6	64.2 ± 10.2	0.656
Sex, n (%)			0.793
Male	31 (77.5)	30 (75.0)	
Female	9 (22.5)	10 (25.0)	
Body weight, kg	66.7 ± 16.9	70.24 ± 12.5	0.284
Body mass index, kg/m^2^	29.9 ± 6.1	28.1 ± 5.6	0.155
Heart rate, beats/min	72.2 ± 10.3	70.1 ± 9.8	0.560
Systolic blood pressure, mm Hg	118.4 ± 9.3	118.5 ± 11.4	0.946
Left ventricular ejection fraction			
Mean value	27.4 ± 4.8	27.3 ± 4.9	0.903
Value of ≤ 30%, n (%)	26 (65.0)	27 (67.5)	0.813
NT-proBNP			
Median value (IQR), pg/mL	1187 (1040–1187)	1183 (1172–1191)	0.938
Value of ≥ 1000 pg/mL, n (%)	30 (75.0)	31 (77.5)	0.793
Cause of heart failure, n (%)			0.823
Ischemic	21 (52.5)	20 (50.0)	
Nonischemic	19 (47.5)	20 (50.0)	

NT-proBNP: N-terminal pro-B-type natriuretic peptide

### Relationship between patients’ NETs, MPO-DNA, and CitH3 levels and venous thrombosis

**Fig. 1:**
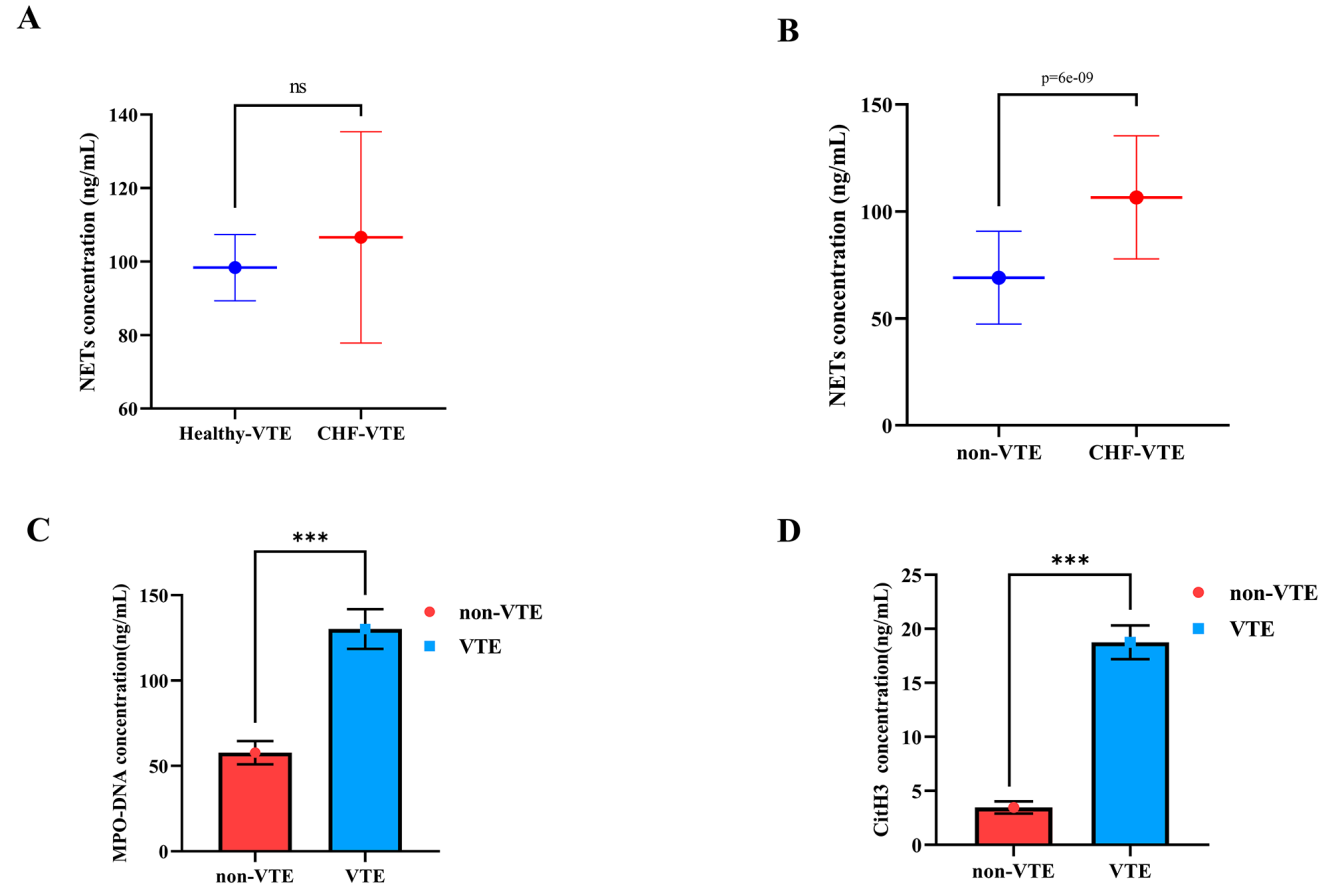
Comparison of NETs, MPO-DNA, and CitH3 levels between patients with and without venous thrombosis (A) NETs levels between Healthy + VTE and CHF + VTE patients; (B) NETs (C) MPO-DNA (D) CitH3 levels between CHF + non-VTE and CHF + VTE patients *** means *P* < 0.001, ns means not statistically significant

We first compared the concentration of NETs between Healthy + VTE patients and CHF + VTE patients. The results showed that the concentration of NETs in the CHF + VTE group was 106.6 ng/mL, which was higher than that in the Healthy + VTE group (98.3 ng/mL), but the difference was not statistically significant (*P* > 0.05), suggesting that the level of NETs may be associated with the presence of CHF (Fig. 1A). Subsequently, the comparison of NETs, MPO-DNA, and CitH3 concentrations in peripheral blood between the VTE and non-VTE groups is shown in Fig. 1B-D. The NETs concentration in the VTE group was 106.6 ng/mL, which was markedly higher than in the non-VTE group (69.1 ng/mL) (*P* < 0.001). The MPO-DNA concentration in the VTE group was 130.20 ng/mL, which was markedly higher than that in the non-VTE group (57.80 ng/mL) (*P* < 0.001). The CitH3 concentration in the VTE group was 18.76 ng/mL, markedly higher than that in the non-VTE group (3.47 ng/mL) (*P* < 0.001).

### Comparison of NETs, MPO-DNA, and CitH3 levels in lesion and peripheral venous blood

**Fig. 2:**
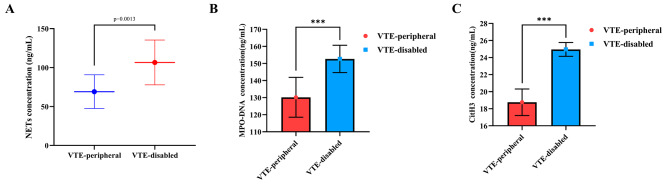
Comparison of NETs (**A**), MPO-DNA (**B**), and CitH3 (**C**) levels in the lesion and peripheral venous blood in CHF patients with venous thrombosis *** means *P* < 0.001

The comparison of NETs, MPO-DNA, and CitH3 concentrations in VTE-disabled vein and VTE-peripheral vein of CHF patients with venous thrombosis is shown in Fig. 2. We found that NETs concentration in the VTE-disabled vein was 125.8 ng/mL, notably higher than in the VTE-peripheral vein (106.6 ng/mL) (*P* = 0.0013). MPO-DNA concentration in the VTE-disabled vein was 152.65 ng/mL, notably higher than in the VTE-peripheral vein (130.20 ng/mL) (*P* < 0.001). CitH3 concentration in the VTE-disabled vein was 24.95 ng/mL, notably higher than in the VTE-peripheral vein (18.76 ng/mL) (*P* < 0.001).

### Relationship between NETs concentrations in the lesion and peripheral venous blood

**Fig. 3:**
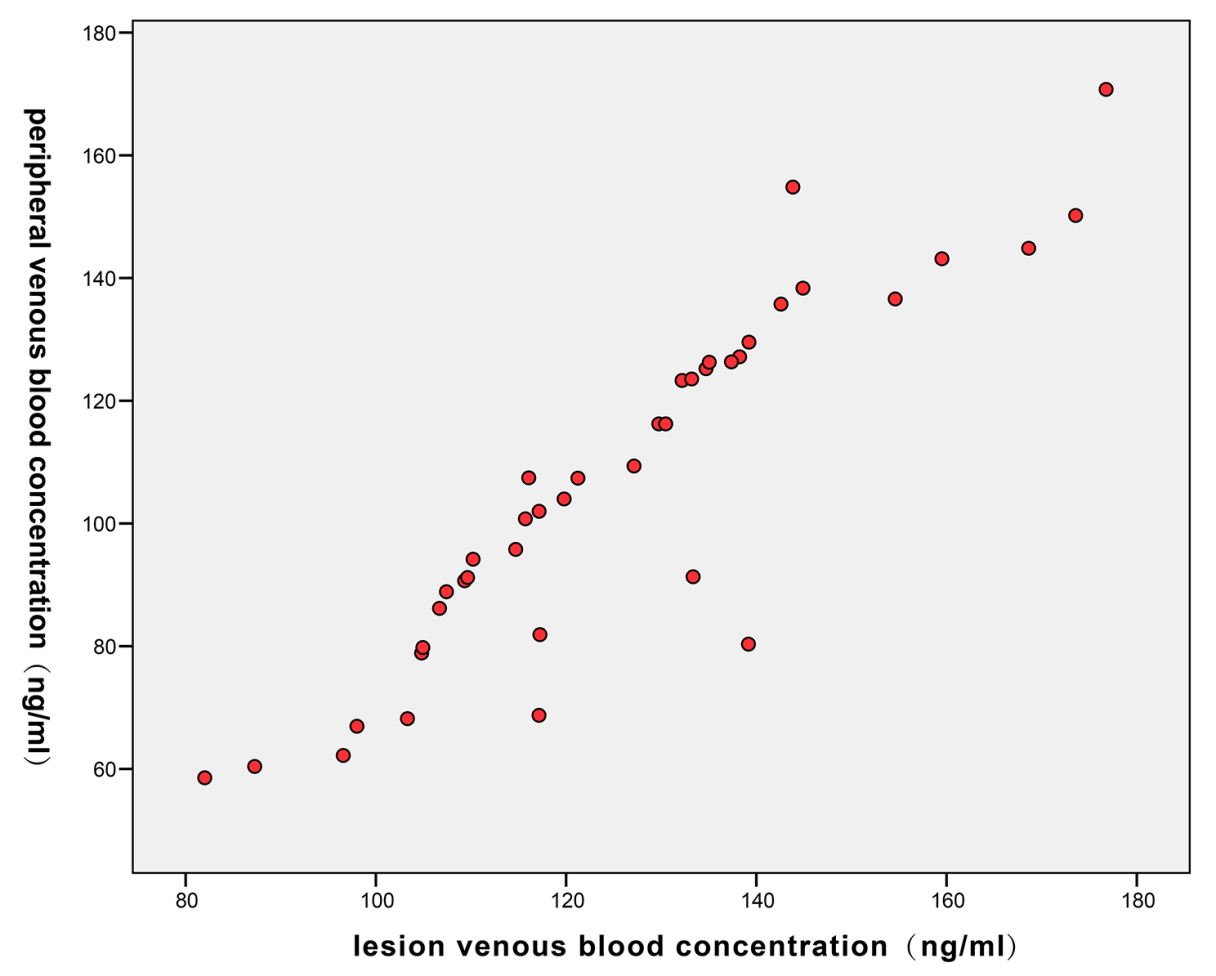
Scatter plot of NETs concentrations in the lesion and peripheral venous blood

Figure 2 showed that the NETs level was significantly higher in the VTE-disabled vein group than in the peripheral vein group, and the correlation between the two was presented by a scatter plot (Fig. 3). In order to explore whether NETs in peripheral venous blood can be used as a diagnostic factor in patients with CHF complicated with venous thrombosis, we first conducted a binary logistics regression analysis. As shown in Table 2, NETs in peripheral venous blood were an independent risk factor (OR = 0.942; 95%CI: 0.917–0.968). Subsequently, a unitary linear regression model was constructed (Table 3). R [2] was 0.818 after model adjustment, and the fitting effect was better. In this model, Y = 1.176X- 41.398, indicating that there was a marked positive correlation between the NETs concentrations in peripheral and lesion venous blood.

**Table 2:** Bivariate logist regression was used to analyze the risk factors of venous thrombosis in patients with chronic heart failure

Participants	OR	95%CI	*P*-value
Gender			
Male	1	-	-
Female	1.782	0.401–7.915	0.448
Age	0.984	0.932–1.039	0.567
Height	1.012	0.971–1.055	0.574
BMI	0.939	0.842–1.047	0.254
Heart rate	1.003	0.942–1.067	0.936
Systolic blood pressure	0.990	0.934–1.049	0.726
Left ventricular ejection fraction	0.982	0.871–1.108	0.771
NT-proBNP	0.999	0.993–1.006	0.827
Cause of heart failure			
Ischemic	1		
Nonischemic	0.818	0.249–2.686	0.740
Peripheral venous blood	0.942	0.917–0.968	0.000

**Table 3:** Regression coefficient table

Model	Unstandardized Coefficients		Standardized Coefficients Beta	*t*	Significance
	B	standard error			
Constant	41.398	11.328		-3.655	0.001
NETs concentration in diseased venous blood	1.176	0.089	0.907	13.260	0.000

### Predictive value of peripheral blood NETs levels for CHF combined with venous thrombosis

**Fig. 4:**
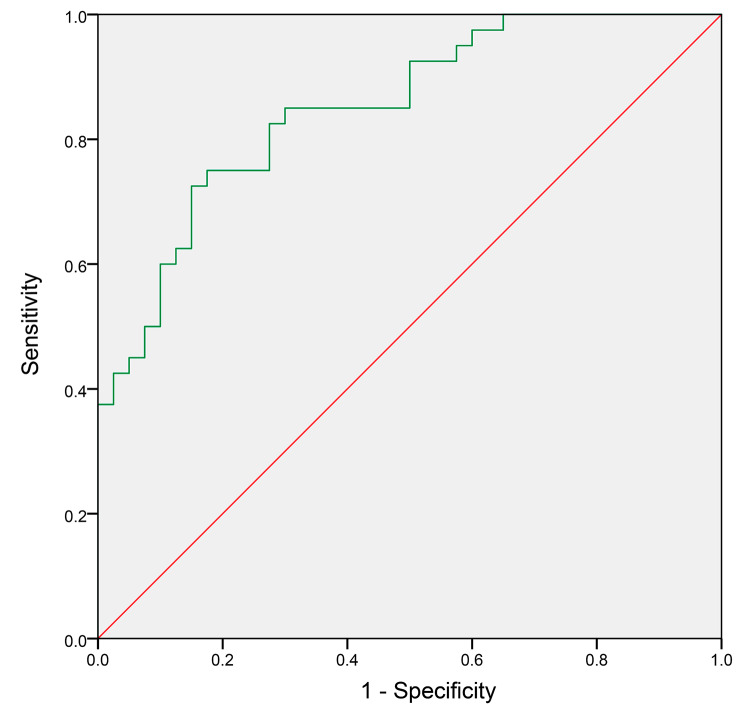
ROC curves for the diagnosis of venous thrombosis in CHF patients by NETs

The results of ROC curve analysis showed that the AUC of peripheral blood NETs for the diagnosis of venous thrombosis in CHF patients was 0.85 (95.0% CI: 0.769–0.931), with a maximum Youden index of 0.575, a sensitivity of 72.5% and specificity of 85.0% at a NETs cut-off value of 88.505 ng/mL, as shown in Fig. 4. Therefore, we concluded that NETs concentration in peripheral venous blood could effectively predict venous thrombosis in CHF patients.

## Discussion

Thrombus is easily formed in CHF due to the complications of vascular wall damage, hypercoagulability, and slow blood flow velocity. Among them, elevated inflammatory factors and hypercoagulability are the primary causes of venous thrombosis in CHF patients [22]. Various diseases are prone to occur in CHF patients with venous thrombosis, such as pulmonary embolism, acute myocardial infarction, and cerebral infarction, which seriously endanger patients’ lives [23]. In current clinical practice, common hematological indicators for detecting thrombosis include coagulation function, fibrinolytic indicator, platelet function, hypercoagulability, etc. In the coagulation function test, increased levels of fibrinogen and factor VII predispose patients to myocardial infarction, and D-dimer is directly associated with cardiac events after myocardial infarction [24, 25]. Plasminogen, tissue type plasminogen activator, and plasminogen activator inhibitor, which represent fibrinolytic capacity, not only regulate the formation and lysis of thrombus in *vivo*, but are also clearly associated with the occurrence of cardiovascular events [26–28]. NT-proBNP and D-dimer are not only crucial biochemical indicators of the severity of illness in CHF patients, but predictors of venous thrombosis in the early and middle stages of hospitalization for patients with heart failure. However, the above methods for predicting venous thrombosis can only be applied after many days of hospitalization [29, 30]. Hematologic indicators are influenced by various factors, easily resulting in missed diagnosis and misdiagnosis. Besides, common imaging methods for the diagnosis of thrombosis include ultrasonography and venography [5, 31]. As a rapid non-invasive test, ultrasonography cannot effectively identify thrombus, because the echogenicity of thrombus is variable and unpredictable, and the fresh clots are anechoic [31]. Additionally, ultrasonography is susceptible to the skill of the operator and may require a change in position during the test, which may be difficult for patients with fractures [32] and ultrasonography cannot predict thrombosis. Venography is the most accurate diagnostic method for VTE. However, it is costly and invasive, and the side effects associated with contrast medium cannot be ignored, so it is used less frequently in clinical practice [5]. Existing diagnostic methods for thrombosis still have technical defects, leaving room for the development of new technology. Therefore, this study provides data support for the development of optimal treatment strategies for early diagnosis, prevention, and timely treatment of venous thrombosis in CHF patients by exploring independent predictive biomarkers of concurrent venous thrombosis in CHF patients.

NETs are now widely recognized as a key factor in thrombosis [17]. As markers of NETs, MPO-DNA is closely related to neutrophil count, and CitH3 is closely correlated with platelet levels. Through a prospective cohort study on the mechanism of immune thrombosis formation in COVID-19 patients with acute respiratory distress, Middleton et al. [33] found that NETs triggering immune thrombus formation could partially illustrate the clinical manifestations of thrombus in COVID-19, and NETs may be therapeutic target. By establishing a mouse model of sepsis and observing the platelet aggregation, thrombin activity, and fibrin clot formation inside NETs in *vivo*, McDonald et al. [34] found that removing NETs dramatically decreases intravascular thrombin activity and platelet aggregation, and improved microvascular perfusion. It was demonstrated that a dynamic NET-platelet-thrombin axis promotes intravascular coagulation and microvascular dysfunction in sepsis. A previous study reported that NETs levels were notably elevated in the serum of symptomatic heart failure patients with or without type 2 diabetes mellitus [35]. However, the expression level and clinical significance of NETs in the peripheral blood of CHF patients with or without venous thrombosis remained unclear. This study found that the concentration of NETs, MPO-DNA, and CitH3 in the VTE group was higher than that in the non-VTE group, suggesting that NETs were expressed highly in the peripheral blood of CHF patients with venous thrombosis. Besides, this study also found that in the VTE group, the concentration of NETs, MPO-DNA, and CitH3 in the lesion vein was higher than that in the peripheral vein. It was indicated that NETs may be involved in the development of venous thrombosis in CHF patients. Similarly, Sharma et al. [36], reported increased levels of neutrophils, neutrophil activation markers (MPO), and NETs in patients with chronic thromboembolic pulmonary hypertension (CTEPH), as well as positive CitH3 results in the fibrin-rich portion of vascular occluded CTEPH patients, suggesting that thrombotic NETs could be a novel therapeutic target to treat thrombosis and prevent the sequelae. However, NETs are not only involved in the formation of venous thrombosis but also associated with arterial thrombosis. Studies have shown that NETs may serve as a potential biomarker for arterial thrombosis in clinical specimens and animal models [37]. Dhanesha et al. [38] found NETs in thrombotic samples from animal models using immunofluorescence staining, and in integrin1α^−/−^ mice, where NET release from neutrophils was reduced, arterial thrombus formation was significantly decreased. Nonetheless, further studies are needed to investigate the role of NETs in the development of arterial thrombosis in patients with CHF.

In addition, the predictive value of NETs in peripheral blood was discussed in this study. ROC analysis showed that NETs concentration levels could effectively determine venous thrombosis in CHF patients. NETs concentrations in peripheral and lesion venous blood were markedly positively correlated. Since NETs were generated in the early stages of thrombosis, we hypothesized that abundant NETs existed inside the mature thrombus [39, 40]. VTE patients included in our study were mostly with old thrombus, therefore NETs concentrations in lesion venous blood were higher than in peripheral venous blood. Although the NETs concentration in lesion veins is higher than that in circulation, it is difficult to identify the formed lesions and the predictive value of NETs for potential thrombosis can be further investigated. Overall, we found that NETs in peripheral blood had clinical value in the diagnosis of venous thrombosis in CHF patients.

In conclusion, our study demonstrated that NETs, MPO-DNA, and CitH3 levels were notably increased in the peripheral blood of CHF patients with venous thrombosis, which showed good diagnostic value for venous thrombosis in CHF patients. However, this study was only conducted at a clinical level with a small sample size, so our findings still need to be validated in a study with a larger sample size. We believe that the NETs levels in peripheral blood provide new insight into the clinical diagnosis of venous thrombosis in CHF patients.

## Data Availability

The datasets used and/or analysed during the current study are available from the corresponding author on reasonable request.
